# Colistin-resistance gene *mcr* in clinical carbapenem-resistant *Enterobacteriaceae* strains in China, 2014–2019

**DOI:** 10.1080/22221751.2020.1717380

**Published:** 2020-01-29

**Authors:** Hong Huang, Ning Dong, Lingbin Shu, Jiayue Lu, Qiaoling Sun, Edward Wai-Chi Chan, Sheng Chen, Rong Zhang

**Affiliations:** a Department of Clinical Laboratory, Second Affiliated Hospital of Zhejiang University, School of Medicine, Hangzhou, People’s Republic of China; b Department of Infectious Diseases and Public Health, Jockey Club College of Veterinary Medicine and Life Sciences, City University of Hong Kong, Kowloon, Hong Kong; c Department of Applied Biology and Chemical Technology, State Key Lab of Chemical Biology and Drug Discovery, The Hong Kong Polytechnic University, Hung Hom, Hong Kong

**Keywords:** Carbapenem-resistant *Enterobacteriaceae*, *mcr-1*, colistin, clinical uses, China

## Abstract

To investigate whether introduction of colistin into the clinical settings selected colistin-resistant CRE, we performed molecular epidemiological study of 1868 CRE strains collected from different geographical locales in China during the period 2014–2019. 1755 (96.18%) isolates carried the carbapenemase genes *bla*_KPC_ and *bla*_NDM_; 14 *Escherichia coli* isolates (0.75%) carrying *mcr-1* and *bla*_NDM_ (MCR-CREC) were also identified. Importantly, the number and relative prevalence of MCR-CREC isolates increased from 5 (0.41%) to 9 (1.38%) after introduction of polymyxin into clinical practice. Consistently, results of genetic analysis indicated that MCR-CREC strains collected before December 2017 were genetically diverse, yet those collected after that date exhibited more closely related genetic profiles, indicating that specific MCR-CREC strains were rapidly selected as a result of increased usage of colistin in clinical settings. The resistance level of MCR-CREC isolates to colistin increased after the introduction of polymyxin into clinical use with the MIC to colistin from <2 mg/L in 80% strains to 2 mg/L in 100% strains. Further dissemination of MCR-CREC strains, which exhibit resistance to the last-line drugs of carbapenems and colistin, is expected to pose a severe threat to human health.

## Introduction

Carbapenem-resistant *Enterobacteriaceae* (CRE) strains pose a serious public health threat worldwide and are associated with high mortality rates [1,2]. *Escherichia coli* and *Klebsiella pneumoniae* are among the most frequently isolated CRE in China. In 2014, the carbapenem resistance rate of these two species was 1.0% and 13.4%, respectively [3,4]. The major mechanisms of carbapenem resistance in CRE include production of carbapenemase, hyper-production of the AmpC β-lactamase, and the combined effect of extended spectrum β-lactamases (ESBLs), porin impermeability, and over-expression of efflux pumps [5]. Carbapenemases commonly produced *Enterobacteriaceae* include KPC, NDM, VIM, IMP, and OXA-48 [6]. A nationwide surveillance of CRE strains conducted in China indicated that the major carbapenemases produced by carbapenem-resistant *K. pneumoniae* (CR*Kp*) and carbapenem-resistant *E. coli* (CR*Ec*) were KPC (74%) and NDM (49%), respectively, and that the predominant sequence types of CR*Kp* and CR*Ec* were ST11 and ST131/ST167, respectively [2,7].

Current treatment options for infections caused by CRE are severely limited. Two β-lactam/β-lactamase inhibitor combinations, ceftazidime/avibactam and ceftolozane/tazobactam, approved for treatment of multidrug resistant gram-negative bacteria abroad, were not available for clinical use in China until June 2019 when ceftazidime–avibactam was officially approved [8]. Treatment regimens against CRE typically rely on last-line antibiotics such as tigecycline and polymyxin [9]. Polymyxin was re-classified as a “critically important” human medicine by WHO in 2012 [10]. In China, polymyxin was approved for use as an injection drug in treatment of bacterial infections in January 2017, and was adopted for clinical use in late 2017 (http://www.mohrss.gov.cn/gkml/zcfg/gfxwj/201702/t20170223_266775.html). Increased usage of polymyxins in clinical settings, however, has led to the emergence of polymyxin-resistant CRE in China and various countries worldwide, especially those carrying the plasmid-borne *mcr* variants [11,12]. In an effort to control polymyxin resistance, the Ministry of Agriculture of China (article number 2428) withdrew colistin from the list of feed additives and growth promoters in November 2016, and this policy was officially enforced in April 2017 [13].

Polymyxin E, also known as colistin, is an important cationic antimicrobial peptide whose clinical potential has been significantly compromised by the global spread of the plasmid-borne colistin resistance genes *mcr* [9,14]. MCR-1 is a phosphoethanolamine transferase firstly reported in late 2015 [14]. A Chinese nationwide epidemiological study revealed a fairly high prevalence of MCR-1-positve *E. coli* among humans (3.7–32.7% among different provinces) [15]. Co-existence of carbapenemase genes such as *bla*_KPC_, *bla*_NDM_, and *bla*_VIM_ and *mcr-1* in CRE isolated from humans, animals (chickens, ducks, and cats), and environmental samples has been reported [16–18]. Nevertheless, according to previous studies, the colistin resistance rate was not particularly high in CR*Kp* (1.1%) and CR*Ec* (2.3%), and carriage of the *mcr-1* gene among CRE remains rare in China [2,19].

To better understand the epidemiological trends and characteristics of MCR-1-producing clinical CRE strains collected before and after polymyxin was approved for use as an antimicrobial agent in clinical practices in China, we conducted extensive and systematic sampling in 24 provinces and municipalities in a span of 5 years (April 2014–April 2019). Findings in this work shall provide essential insight into development of effective strategies for worldwide control of *mcr-1*-carrying CRE and reduce the rate of untreatable hospital infections.

## Materials and methods

### Study design

To investigate the carriage of CRE, especially *mcr-1*-carrying CRE among hospital patients, we performed a cross-sectional multi-center study on samples collected from 1 January 2014 to 30 April 2019. Non-duplicated *Enterobacteriaceae* strains that exhibited carbapenem resistance phenotype (meropenem MIC ≥ 4 µg/mL) were collected from infection sites and clinical specimens of the patients including blood, urine, sputum, bile, hydrothorax, ascites, and various other specimens. All consecutive CRE isolates in the selected hospitals were stored and tested in the study period. A total of 1868 strains were collected from hospitals located in 24 provinces and municipalities in China including Anhui, Beijing, Fujian, Gansu, Guangdong, Guangxi, Guizhou, Hainan, Hebei, Henan, Hubei, Hunan, Jilin, Jiangxi, Liaoning, Jiangsu, Shandong, Shanxi, Shaanxi, Shanghai, Sichuan, Tianjin, Xinjiang, and Zhejiang (Table S1). These areas cover a population of 1.23 billion (∼90%) in China. One representative hospital, namely the largest general hospital in each location, was chosen for sample collection. Polymyxin has been applied in all these hospitals in 1 December 2017. All strains were subjected to species identification using the matrix-assisted laser desorption/ionization time-of-flight mass spectrometry (Bruker Daltonik GmbH, Bremen, Germany).

### Gene screening and antimicrobial susceptibility testing

Carriage of carbapenem resistance genes (*bla*_VIM_, *bla*_IMP_, *bla*_KPC_, *bla*_NDM_, and *bla*_oxa-48-like_) and the colistin resistance gene *mcr-1* to *mcr-9* in all CRE isolates were screened by PCR, using methods described previously [14,20–22]. The genetic identity of the PCR products was validated using Sanger sequencing. All *mcr-1*-positive CRE strains were subjected to antimicrobial susceptibility testing against 15 commonly used antibiotics, using micro-broth dilution method. The minimum inhibitory concentrations (MICs) for carbapenems (imipenem, meropenem, ertapenem), cephems (cefepime, ceftazidime, cefotaxime, cefmetazole), fluoroquinolone (ciprofloxacin), β-lactam/β-lactamase combinations (piperacillin–tazobactam, cefoperazone–sulbactam (2:1), ceftazidime–avibactam), aminoglycoside (amikacin), and monobactam (aztreonam) were interpreted according to the Clinical and Laboratory Standards Institute (CLSI) guidelines [23]. Breakpoints for colistin and tigecycline were interpreted according to the European Committee on Antimicrobial Susceptibility Testing (EUCAST) criteria [24].

### Whole-genome sequencing and bioinformatics analysis

All *mcr-1*-positive CRE isolates were subjected to whole-genome sequencing. Genomic DNA of each test isolate was extracted from overnight cultures using the PureLink Genomic DNA Mini Kit (Invitrogen, Carlsbad, CA, USA). Genomic libraries were prepared with an insert of a size of ∼350 bp by the TruSeq DNA PCR-free Sample Preparation Kit (Illumina Inc., San Diego, CA, USA), following the manufacturer’s instructions and sequenced on the Illumina HiSeq 2500 platform (Annoroad Biotech Co.). Raw reads were trimmed with Trimmomatic to remove low-quality sequences and adaptors [25]. *De novo* assembly was conducted with SPAdes Genome Assembler v3.12.1 [26]. Assembled genome sequences were annotated with RAST [27]. Mobile antibiotic resistance genes were identified with ResFinder 2.1 [28]. Plasmid replicons were analysed using PlasmidFinder [29]. Insertion sequences (ISs) were identified using ISfinder [30]. Multilocus sequence typing and serotyping were conducted using MLST v2.11 and ECTyper v0.8.1, respectively [31,32]. Plasmid alignment was conducted using BRIG [33]. The harvest suite v.1.2 was used to remove recombination sequences and conduct the phylogenetic analysis using the assembled genome sequences as input and the genome sequence of strain SC-6 was used as a reference [34]. Phylogenetic tree was visualized and modified using iTOL v4 [35]. All draft genome sequences have been deposited in GenBank under BioProject accession number PRJNA558538.

### Quantitative real-time PCR

Total RNA of *mcr-1*-positive strains was extracted using the RNeasy Mini kit (Qiagen, Hilden, Germany). Residual DNA was removed from the total RNA samples, and cDNA was synthesized with a PrimeScript RT Reagent Kit with gDNA eraser (TAKARA, Dalian, China) following the manufacturer’s instruction. Quantitative RT-PCR assay was performed using the SYBR® Premix Ex Taq™ II (Tli RNaseH Plus) kit (TAKARA) with primers published previously [36]. The 23S rRNA gene was used as the endogenous reference gene. *Escherichia coli* ATCC 25922 with a colistin MIC of 0.25 μg/mL was used as the reference strain. Relative expression level of the *mcr-1* gene was obtained by the ΔΔCT analysis method.

### Statistical analysis

The differences of *mcr-1* carrying rate before and after December 2017 were assessed by Chi-Square Tests on IBM SPSS Statistics 20, and the selected specific chi-square test type depends on the expected count and the sample size of valid cases. Besides, the *p* values of <0.05 were considered to be statistically significant.

## Results

### Overview of the CRE strains

**Figure 1.**
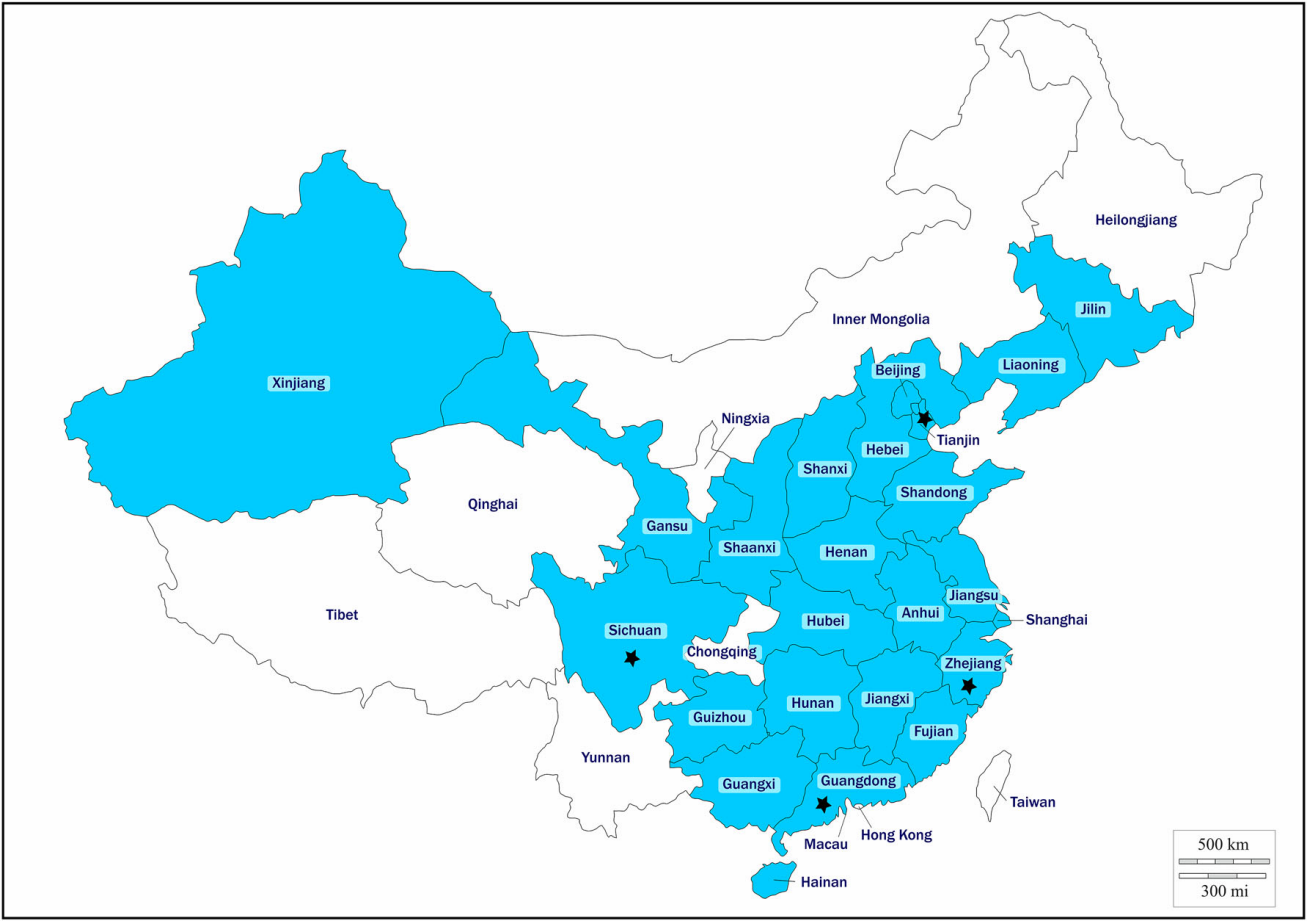
Distribution of *mcr-1* in carbapenem-resistant *Enterobacteriaceae* strains in China. *mcr-1*-producing isolates were denoted with stars. The provinces included in this study are shaded.

A total of 1868 non-duplicated CRE isolates were obtained from hospitals in 24 provinces and municipalities in China, including 1134 *K. pneumoniae* isolates, 376 *E. coli* isolates, 184 *Enterobacter cloacae* isolates, 41 *Serratia marcescens* isolates, 45 *Enterobacter aerogenes* isolates, 31 *K. oxytoca* isolates, 29 *Citrobacter freundii* isolates, and 28 other isolates (*C. koseri*, *C. braakii*, *Raoultella ornithinolytica*, *Providencia rettgeri*, and *Proteus mirabilis*) (Table 1). The number of strains isolated before and after 21 February 2017 were 1215 and 653, respectively. PCR assay indicated that 1755 (96.18%) isolates carried the carbapenemase genes *bla*_KPC_ and *bla*_NDM_, and that 14 isolates (0.75%) were positive for *mcr-1*. No strain positive for other *mcr* variants was detected. The 14 *mcr-1* carrying isolates, all identified as *E. coli* and termed MCR-CREC hereafter, were isolated from sputum, blood, drainage, secretion, pus, and throat swab specimens (Table 2). The *bla*_NDM_ gene, but not other carbapenem resistance genes, were identified in all the MCR-CREC isolates. Among these 14 *bla*_NDM_ genes, 2 (14.29%) were *bla*_NDM-4_, 11 (78.57%) were *bla*_NDM-5_, and 1 (7.14%) was *bla*_NDM-9_. The MCR-CREC isolates were recovered from four provinces or regions, namely Zhejiang (*n* = 9, 64.29%), Tianjin (*n* = 2, 14.29%), Guangdong (*n* = 2, 14.29%), and Sichuan (*n* = 1, 7.14%) (Figure 1, Table 2). The number and proportion of MCR-CREC isolates among the test strains were found to increase from 5 (0.41%) to 9 (1.38%) after introduction of polymyxin into clinical practice, such increase was statistically significant (*P *< 0.05). MCR-CREC were detectable in all the four provinces mentioned above before 1 December 2017; however, eight of the nine (88.9%) MCR-CREC strains isolated thereafter were collected from Zhejiang province, indicating that a rather localized dissemination of MCR-CREC strains had occurred, presumably due to antibiotic selection.

**Table 1.** Distribution of mcr-1 in CREs during 2014–2019.

Strain species	No. of strains (1 April 2014–20 February 2017)						No. of strains (21 February 2017–30 April 2019)						*P*-value*
	CRE	*bla*_KPC_ positive	*bla*_NDM_ positive	CPE	*mcr-1*	*mcr-1* carrying rate (%)	CRE	*bla*_KPC_ positive	*bla*_NDM_ positive	CPE	*mcr-1*	*mcr-1* carrying rate (%)	
*K. pneumoniae*	740	554	121	705	0	0	394	309	32	384	0	0	–
*E. coli*	235	85	132	221	5	2.13%	141	62	73	137	9	6.38%	**0.035**
*Enterobacter cloacae*	132	19	81	105	0	0	52	9	39	52	0	0	–
*Serratia marcescens*	21	6	14	21	0	0	20	7	0	7	0	0	–
*Enterobacter aerogenes*	26	6	16	26	0	0	19	15	1	16	0	0	–
*Citrobacter freundii*	21	2	13	21	0	0	8	3	5	8	0	0	–
*K. oxytoca*	24	7	10	24	0	0	7	2	3	5	0	0	–
Other species	16	7	7	12	0	0	12	6	5	11	0	0	–
Total	1215	686	394	1135	5	0.41%	653	413	158	620	9	1.38%	**0.042**

CRE, carbapenem-resistant Enterobacteriaceae; CPE, carbapenem-producing Enterobacteriacease.

*Differences between groups were assessed by Pearson chi-square and continuity correction. *P*-value in bold indicates statistical significance.

**Table 2.** Strain source, susceptibility to commonly used antibiotics and *mcr-1* expression levels of *mcr-1*-positive isolates**.**

Bacterial	Year	provinces	specimen	MLST	Serotype	*mcr-1*- plasmid	*bla*_NDM_- plasmid	MIC (mg/L)															ΔΔCt expression** (average ± StdDev)
								IPM	MEM	ETP	CMZ	CAZ	CTX	TZP	SCF	CAV	FEP	PE	TGC	CIP	AK	ATM	
**1 April 2014–20 February**																							** **
TJ-2	2015	Tianjin	sputum	167	O89:H9	IncX4	Na*	32	64	128	>128	>128	>128	>256/4	>256/4	>64/4	>64	≤0.5	≤0.25	>32	>128	32	2.279 ± 0.547
GD133	2015	Guangdong	blood	10	O89:H10	IncHI2	IncX3	16	16	32	16	>128	>128	256/4	256/4	>64/4	64	1	≤0.25	32	≤4	16	227.873 ± 41.474
GD119	2015	Guangdong	sputum	10	O89:H10	IncHI2	IncX3	16	64	64	>128	>128	>128	>256/4	>256/4	>64/4	>64	≤0.5	≤0.25	>32	≤4	>128	289.955 ± 55.608
SC-6	2015	Sichuan	sputum	167	O89:H9	IncX4	IncX3	32	128	128	>128	>128	>128	>256/4	>256/4	>64/4	>64	2	≤0.25	32	≤4	16	107.323 ± 17.276
C34	2015	Zhejiang	Secretion	5229	O177:H51	IncX4	IncX3	32	128	128	>128	>128	>128	>256/4	>256/4	>64/4	>64	≤0.5	≤0.25	>32	>128	>128	Na. *
**21 February 2017–30 April 2019**																							** **
TJ-71	2017	Tianjin	Drainage	617	O89:H9	IncI2	IncFII	8	32	64	>128	>128	>128	>256/4	>256/4	>64/4	>64	2	≤0.25	32	>128	>128	107.539 ± 19.591
363	2017	Zhejiang	sputum	4380	O116:H9	IncHI2A/HI2	Na*	16	32	32	64	>128	>128	256/4	>256/4	>64/4	64	2	≤0.25	32	≤4	≤4	133.119 ± 15.133
404	2017	Zhejiang	sputum	23	O78:H9	IncHI2	IncX3	8	16	32	16	>128	>128	>256/4	256/4	>64/4	64	2	≤0.25	≤1	≤4	≤4	448.233 ± 116.063
34	2017	Zhejiang	sputum	10	O60:H12	IncX4	IncX4	8	16	32	16	>128	>128	128/4	256/4	>64/4	64	2	≤0.25	≤1	≤4	≤4	522.624 ± 84.262
76	2017	Zhejiang	Pus	2179	O9:H9	IncHI2	IncX3	16	32	32	8	>128	>128	128/4	256/4	>64/4	32	2	≤0.25	16	≤4	≤4	246.972 ± 39.069
182	2017	Zhejiang	Throat swab	189	O37:H21	IncY	IncX3	8	16	32	32	>128	>128	128/4	256/4	>64/4	64	2	≤0.25	16	≤4	≤4	107.892 ± 21.309
362–1	2017	Zhejiang	blood	4380	O116:H9	IncHI2A/HI2	Na*	8	8	16	128	>128	>128	128/4	128/4	>64/4	16	2	≤0.25	≤1	≤4	32	189.66 ± 43.697
shaw79	2019	Zhejiang	sputum	617	O89:H9	IncI2	IncFII	8	16	64	>128	>128	>128	>256/4	>256/4	>64/4	>64	2	≤0.25	>32	≤4	≤4	194.778 ± 43.785
sz16	2019	Zhejiang	blood	297	O_unknown_:H9	IncI2	IncFII	8	32	32	>128	>128	>128	>256/4	>256/4	>64/4	>64	2	≤0.25	8	≤4	>128	190.368 ± 35.552

IMP, imipenem; MEM, meropenem; ETP, ertapenem; CMZ, cefmetazole; CAZ, ceftazidime; CTX, cefotaxime; TZP, piperacillin/tazobactam; SCF, cefoperazone/sulbactam; CAV, ceftazidime/avibactam; FEP, cefepime; PE, polymyxin E; TGC, tigecycline; CIP, ciprofloxacin; AK, amikacin; ATM, aztreonam.

*Na, not applicable; **ΔΔCt here was used to describe the relative *mcr-1* gene expression levels in the strains.

### Antimicrobial susceptibility of MCR-CREC and expression level of mcr-1

The 14 MCR-CREC isolates were all resistant to carbapenems but were susceptible to tigecycline. The proportion of MCR-CREC isolates that were resistant to colistin, ciprofloxacin, amikacin, and aztreonam were 71.4%, 78.6%, 21.4%, and 57.1%, respectively (Table 2). Among the five MCR-CREC strains isolated before 1 December 2017, only one (20%) was resistant to colistin (MIC = 2 mg/mL), one (20%) exhibited intermediate resistance to colistin (MIC = 1 mg/mL), and the other three (60%) remained susceptible to colistin (MIC ≤0.5 mg/mL). However, all nine MCR-CREC strains isolated after 1 December 2017 were resistant to colistin, exhibiting an MIC of 2 mg/mL (Table 2, Fig S1) indicating that colistin MICs for *mcr-1*-positive CREC increased after clinical use of colistin.

To test whether the clinical application of colistin selected the expression of *mcr-1*, relative expression levels of *mcr-1* was measured by qRT-PCR assays (Table 2, Fig S2). Strain C34 was excluded in the qPCR experiment due to the similar resistance profiles of strains C34 and TJ-2. Among the 13 strains tested, TJ-2 isolated in 2015 exhibited the lowest *mcr-1* expression level. The expression levels of *mcr-1* gene in other strains isolated before 1 December 2017 were 47, 100, and 127 folds of that of TJ-2. That of strains isolated after 1 December 2017 were 47 (2 strains), 58, 83 (2 strains), 85, 108, 197, and 224 folds of strain TJ-2. With these data, we could not determine whether the use of colistin select the expression of *mcr-1*. Despite strains isolated after 1 December 2017 exhibited identical susceptibility phenotypes to colistin (MIC = 2 mg/L), the expression levels of *mcr-1* varied dramatically among strains, indicating the expression of *mcr-1* was potentially under regulation of other genetic components, which warrants further investigation.

### Genetic background of MCR-CREC

**Figure 2.**
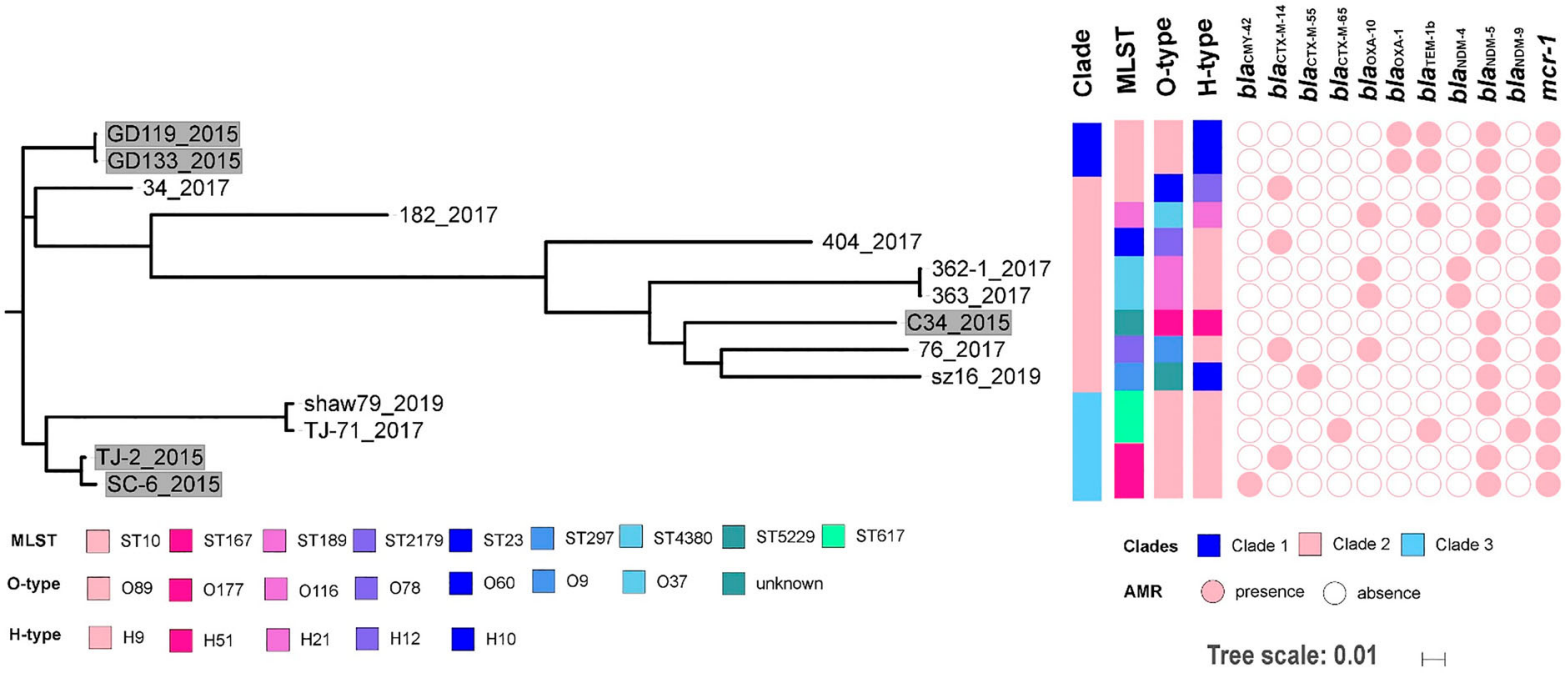
Mid-point rooted phylogeny of *mcr-1*-bearing *E. coli* strains. (A) Strains isolated before 1 December 2017 were labelled with grey backgrounds. MLST, O and H serotypes, the presence of antimicrobial resistance genes (AMR) encoding ESBLs, carbapenemases, and MCR-1 were plotted. Year of isolation for each strain was denoted after the dash line.

The 14 MCR-CREC isolates were allocated to nine ST types and different serotypes (eight O types and five H types, Table 2, Figure 2). None of these ST types was dominant, but ST167 (*n* = 2), ST617 (*n* = 2), ST4380 (*n* = 2), and ST10 (*n* = 2) were more prevalent than the other sequence types. A total of 60,534 SNPs (minimum: 34; maximum: 29698) were identified among the core genomes of these isolates, on the basis of which the isolates could be allocated into least three major lineages (Fig S3). The first clade includes two ST10 *E. coli* isolates (GD119, GD133) both isolated before 1 December 2017. The second clade represents a major branch of *mcr-1*-bearing carbapenem-resistant *E. coli*, which comprised eight isolates (34, 182, 404, 362-1, 363, C34, 76, sz16) of seven sequence types, with seven out of the eight isolates being isolated after 1 December 2017. The third clade includes four isolates, two isolated before (*n* = 2, ST167) and two after (*n* = 2, ST617) December 2017. The data indicated that MCR-CREC strains were genetically diverse and covered all three genetic clades before the use of colistin in clinical setting with only one strain belonging to clade II, yet most of the strains after the use of colistin in clinical setting were selected in clade II. When looked at the serotypes, four strains of O89 belonging to clade I and III were isolated before use of colistin, while more diverse O types in clade II were found in *mcr-1*-positive CREC after the use of colistin.

Multiple antimicrobial resistance genes, including ESBLs (*bla*_CMY_, *bla*_CTX_, *bla*_TEM_, and *bla*_OXA_), carbapenemase gene *bla*_NDM_ and the *mcr-1* gene, were detectable among the test isolates (Figure 2).

To further investigate the role of MCR-CREC strains in dissemination of the *bla*_NDM_ and *mcr-1* genes, we analysed the draft whole-genome sequence of the 14 MCR-CREC strains and found that the *mcr-1* gene was located in contigs ranging from 2 to 109 kb. The *mcr-1*-bearing contigs in MCR-CREC aligned well to different plasmids, including the 97 kb IncY pHYEC7-MCR1 plasmid (accession: KX518745, *n* = 1), the 169 kb IncHI2A/HI2 pHNSHP47 plasmid (accession: MF774186, *n* = 2), the 251 kb IncHI2 pHNSHP45-2 plasmid (accession: KU341381, *n* = 4), the 33 kb IncX4 pESTMCR plasmid (accession: KU743383, *n* = 3), and the 63 kb IncI2 pOM97-mcr plasmid (accession: KY693674, *n* = 3) (Fig S4). IncX4 and IncHI2 types of *mcr-1*-bearing plasmids were detected in these CREC isolates, while three more plasmids including IncI2, IncHI2A/HI2, and IncY were detected in isolates after the use of colistin (Table 2). BLAST of the *bla*_NDM_ genes in the draft genomes of these strains indicated that the gene was located on contigs ranging from 2 to 43 kb. Genomic contents of the 14 strains aligned well to the 46 kb IncX3 plasmid pNDM-EC36 (accession: MG591703, *n* = 7) and the 116 Kb IncFII plasmid pEC1107-NDM-116 K (accession: MG601057, *n* = 3), separately (Fig S5). Due to poor assembly quality, contigs carrying *bla*_NDM_ in three isolates (TJ-2, 221, 362-1, and 363) were too short to resolve the picture of the plasmid harbouring *bla*_NDM_. Besides, co-existence of *mcr-1* and *bla*_NDM_ in one contig was detected in one isolate, 34, which was isolated before December 2017. The contig bearing both genes aligned well to the 48 kb IncX4 plasmid pCQ02-121 (accession: KU647721, Fig S6). For isolates carrying known types of *bla*_NDM_-bearing plasmids, all isolates before the use of colistin carried incX3 types of plasmids, while IncFII and IncX4 type of plasmid were found in addition to IncX3 type in CREC isolates after the use of colistin (Table 2).

## Discussion

Since the discovery of the plasmid-mediated polymyxin resistance gene *mcr-1* at the end of 2015, mobile colistin resistance determinants have attracted worldwide attention [14]. To date, nine different *mcr* variants, namely *mcr-1* to *mcr-9*, have been documented [22]. These *mcr* genes have been disseminated worldwide and were reported to coexist with many different resistance genes, especially carbapenem resistance genes (*bla*_NDM_, *bla*_KPC_, etc.), resulting in the emergence of veritable “superbugs” which pose severe threats to human health [37–41]. To our knowledge, this study was the first nationwide surveillance report comparing the carriage of *mcr-1* among CRE before and after use of polymyxin in clinical settings.

The prevalence of colistin resistance was relatively low in *Enterobacteriaceae* strains in China and many other countries [42,43]. In this study, although the *mcr-1* carriage rate was not particularly high among CRE (0.75%), our data show that the percentage of CRE isolates that carry *mcr-1* increased dramatically from 0.41% to 1.38% after polymyxin was adopted for clinical use, indicating the use of colistin prompted the emergence and spread of MCR-CRE in China. Most MCR-CREC strains (*n* = 9) were isolated from Zhejiang province, probably due to the relatively high antimicrobial resistance rates in Zhejiang and a large proportion of strains (28%, 524/1868) was collected from Zhejiang province. Alarmingly, we found the resistance level of MCR-CREC isolates to colistin increased after the introduction of polymyxin into clinical use with the MICs to colistin increasing from <2 mg/L in 80% strains to 2 mg/L in 100% strains. The quantification of the *mcr-1* gene was tricky due to strain differences, thus we could not conclude whether the use of colistin have selected the expression of *mcr-1* in the CREC strains. But the worrying scenario of that *mcr-1* as well as the colistin resistance level increased within a short period of time (2014–2019) is a reminder that we need to take urgent actions.

The *bla*_KPC-2_ and *bla*_NDM_ was responsible for phenotypic resistance in 58% and 32% of the CRE strains in China, respectively [2]. Co-existence of *mcr* genes and different carbapenem resistance genes such as *bla*_KPC-3_, *bla*_NDM-5_, and *bla*_NDM-16_ have been sporadically reported in Enterobacteriaceae isolated from patients from different continents, among which *E. coli* was the most reported hosts [44–46]. Consistently, all *mcr-1*-positive CRE strains in this study belonged to *E. coli*, indicating *E. coli* was the major reservoir of such resistance genes. A previous study indicated acquisition of *mcr-1* or *bla*_NDM-5_ plasmid did not lead to considerable fitness costs, highlighting the potential for dissemination of *mcr-1* and *bla*_NDM-5_ in Enterobacteriaceae [47]. In this study, *mcr-1* and *bla*_NDM-5_ are the most dominant (78.6%, 11/14) combination among MCR-CREC strains. *Escherichia coli* isolates carrying both *mcr-1* and *bla*_NDM-5_ also have disseminated among healthy people in various regions of China [48]. The pattern of transmission of resistance genes between patients and healthy individuals should also be carefully monitored. More diverse *bla*_NDM_ variants including *bla*_NDM-4_ and *bla*_NDM-9_ emerged in the clinical settings after the use of polymyxin, suggesting an alarming evolutionary trend of MCR-CREC strains.

Several different *E. coli* ST isolates such as ST131, ST405, ST167, ST206, ST648, ST10, ST617, and ST156 have been reported to carry both *bla*_NDM_ variants and *mcr* genes [47,49,50]. In this study, three and seven *E. coli* ST types were detected before and after the use of colistin, respectively. The serotypes of the MCR-CREC strains were more diverse after use of colistin. These findings suggested the application of colistin in clinical settings expanded the host range of *E. coli* strains carrying both *bla*_NDM_ variants and *mcr-1* genes. *bla*_NDM_ variants and *mcr* determinants are predominantly encoded by mobile genetic elements such as conjugative plasmids, transposons, and integrons, which are readily transferable [51]. The major plasmid types carrying *mcr-1* published to date included IncX4, IncI2, IncHI2, IncY [52,53]. Diverse plasmid types carrying *bla*_NDM_ variants have been reported, including IncX3, IncFII, IncH, and IncA/C [54–57]. The type of plasmids carrying *bla*_NDM_ variants and *mcr-1* both became more diverse under colistin treatment in clinical settings, indicating that colistin treatment prompted the transmission of diverse resistance plasmids among *E. coli* isolates. Besides, a strain (*E. coli* 34) with IncX4 plasmid carrying both *bla*_NDM-5_ and *mcr-1* genes was isolated after December 2017. Co-transfer of *bla*_NDM_ and *mcr-1* by a single plasmid has been rare since the discovery of *mcr-1*, with only one case reporting a plasmid (pCQ02-121) similar to the one in this study generated from genetic recombination between the IncX3 *bla*_NDM-5_-carrying and the IncX4 *mcr-1*-carrying plasmid [41]. pCQ02-121 was from an ST156 *E. coli* strain isolated from a pet cat in China in 2015, and in this study, *E. coli* 34 was an ST10 strain isolated from a human carrier, suggesting the transmission potential of pCQ02-121-like plasmids within different host strains and among different carrier organisms such as humans and companion animals [41]. Heightened efforts are needed to control the dissemination of pCQ02-121-like plasmids. The potential of *E. coli* isolates to acquire plasmids and mobile elements carrying *bla*_NDM_ and *mcr-1* genes rendered them to become “superbugs” resistance to last-line antibiotics which posed a grave threat to human health.

## Conclusions

This is the first comprehensive study which investigated the carriage of *mcr-1* by CRE among individuals in the clinical settings. CRE strains positive for *mcr-1* were all *E. coli* with diverse sequence types and serotypes. The official issue of polymyxin in hospitals in February 2017 lead to a significant increase in MCR-CREC isolates and elevated resistance profiles to polymyxins. NDM was the major carbapenemase carried by MCR-CREC, with NDM-5 being the most dominant type. Genomic analysis indicated the *mcr-1* and *bla*_NDM_ genes were carried by different plasmids which are highly transmissible. Due to the high prevalence of CRE and increasing carriage of MCR-CREC isolates among patients in the clinical settings, urgent actions should be taken to prevent the dissemination of such pathogens in high-risk patients.

## Supplementary Material

Supplemental Material
