# Supplementary material for: Colistin-resistance gene mcr in clinical carbapenem-resistant Enterobacteriaceae strains in China, 2014–2019
Source: Emerg Microbes Infect. 2020 Jan 29;9(1):237–45. doi: 10.1080/22221751.2020.1717380 (PMC7034111; doi:10.1080/22221751.2020.1717380)
Supplement: Supplemental Material [file TEMI_A_1717380_SM5693.zip › Supplementary+materials+R2.docx]

Supplementary materials

**Carriage rate of the colistin-resistance gene *mcr-1* in clinical carbapenem-resistant *Enterobacteriaceae* strains in China: 2014-2019**

Hong Huang ^1, #^, Ning Dong ^2 ,#^, Linbin Shu^1^, Jiayue Lu ^1^, Qiaolin Sun ^1^，Edward Waichi Chan^3^, Sheng Chen ^2*^, Rong Zhang ^1,*^

^1^Department of Clinical Laboratory, Second Affiliated Hospital of Zhejiang University, School of Medicine, Hangzhou 310009, China;

^2^Department of Infectious Diseases and Public Health, Jockey Club College of Veterinary Medicine and Life Sciences, City University of Hong Kong, Kowloon, Hong Kong.

^3^State Key Lab of Chemical Biology and Drug Discovery, Department of Applied Biology and Chemical Technology, The Hong Kong Polytechnic University, Hung Hom, Hong Kong;

#Contribute equally to this work.

*Correspondence to Sheng Chen (shechen@cityu.edu.hk) and Rong Zhang (zhang-rong@zju.edu.cn)

**Keywords:** Carbapenem-resistant *Enterobacteriaceae*，mcr-1, colistin, clinical uses, China





**Supplementary Figure S1. MIC of colsitin for MCR-CREC strains isolated before and after the use of colisitn in clinical setting.** The break point of colsitin is 2.0µg/ml in *E. coli.*


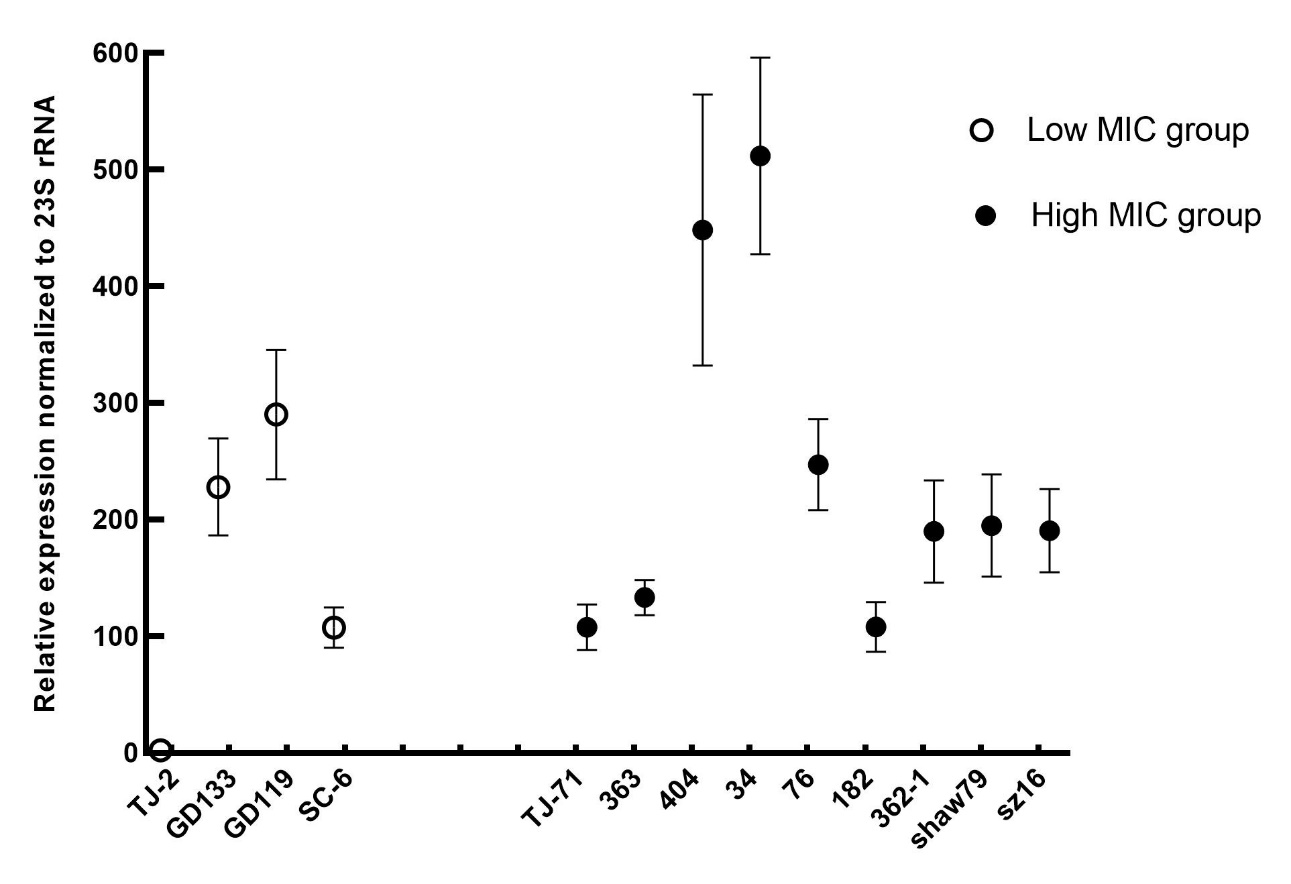


**Supplementary Figure S2. Relative expression levels of *mcr-1* in MCR-CREC strains.** Low and high MIC group represented strains isolated before and after December 1, 2017, respectively. The relative expression levels of *mcr-1* in each strain was compared with that of strain TJ2 which exhibited a colistin MIC of ≤0.5 μg/mL.

**
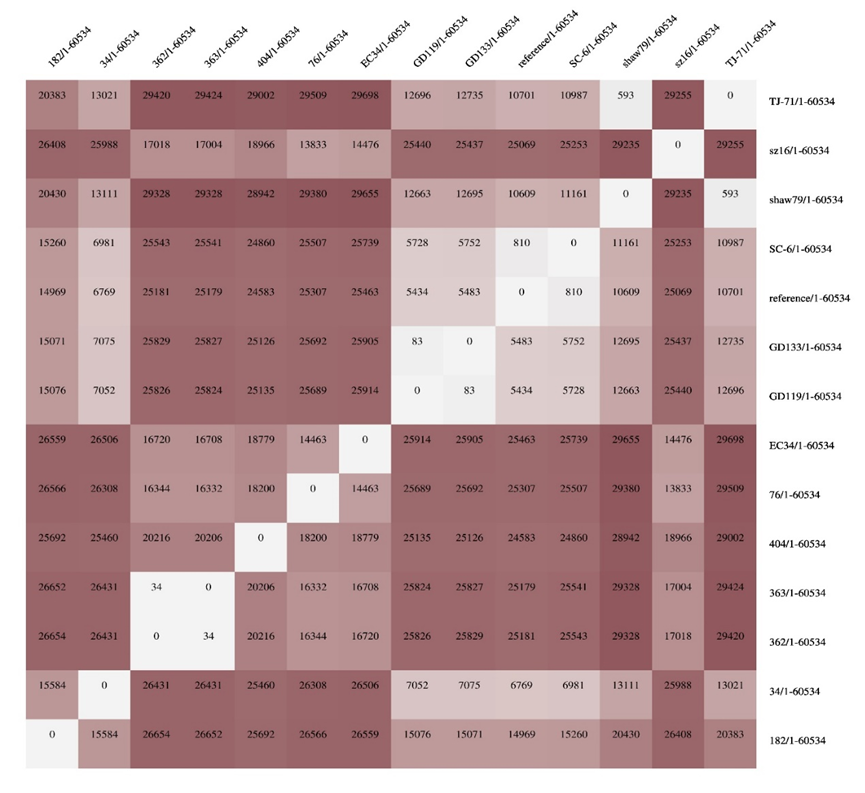
**

**Supplementary Figure S3. Matrix of SNP pair counts among clinical MCR-CREC strains.** Number of SNP was calculated by comparing the core genome sequences to the reference (TJ-2) genome. Background colors represent different number of pairwise SNPs, ranging from a minimum of 34 (white background) to a maximum of 29698 (mahogany).


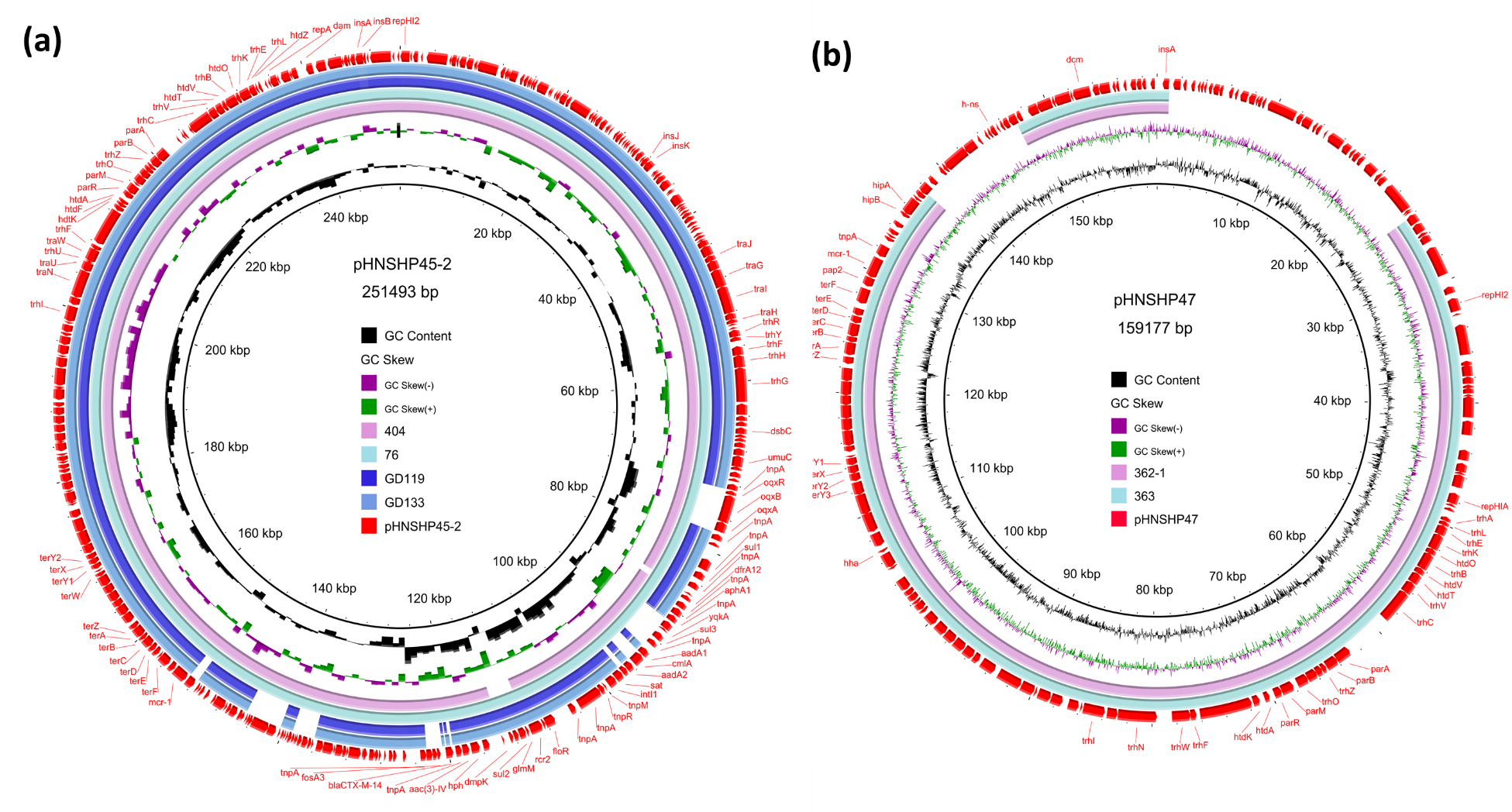


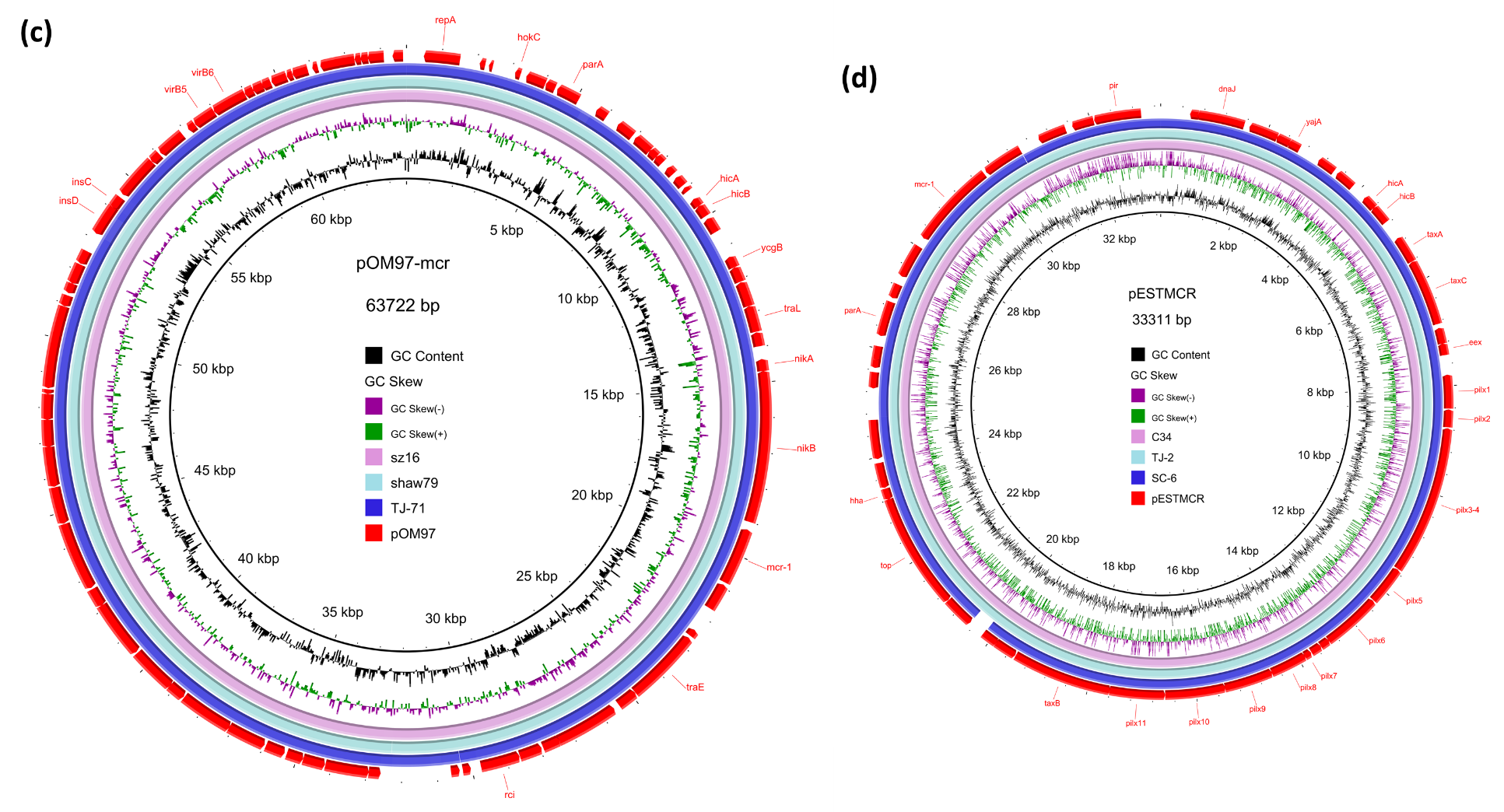


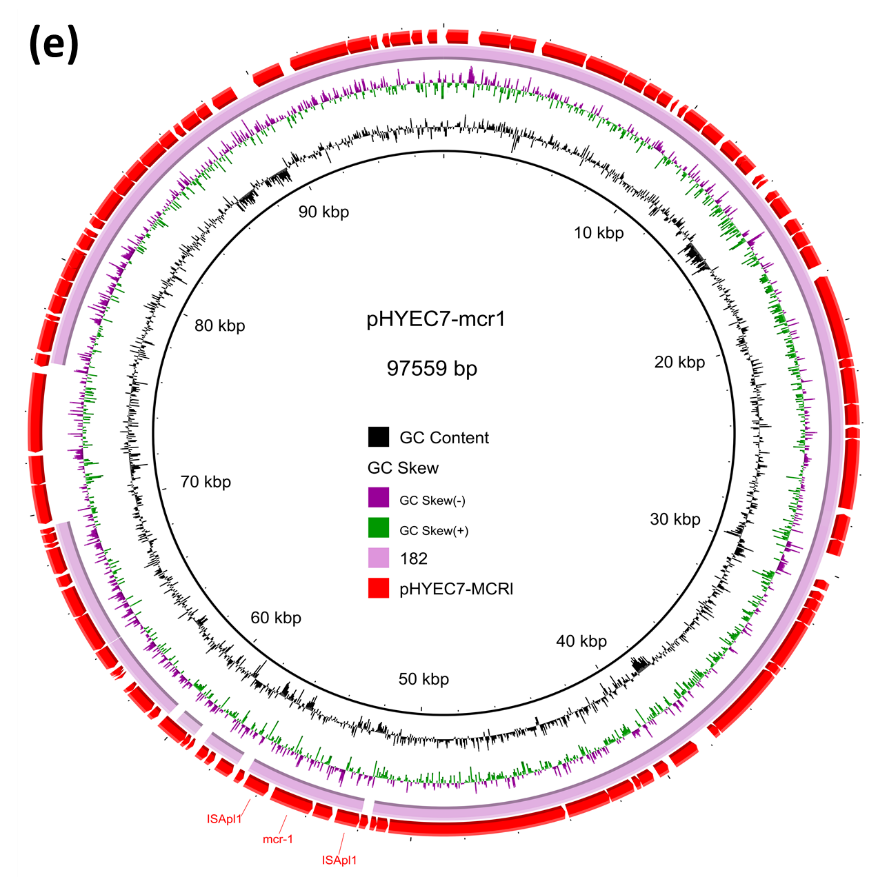


**Supplementary Figure S4. Circular alignment of *mcr*-1-bearing plasmids.** The assembled contigs in this study was mapped to the prototype plasmids. The following plasmid types were included: (a) pHNSHP45-2-like IncHI2 plasmid; (b) pHNSHP47-like IncHI2A/HI2 plasmid; (C) pOM97-mcr-like IncI2 plasmid; (D) pESTMCR-like IncX4 plasmid; (E) pHYEC7-MCR1-like IncY plasmid.


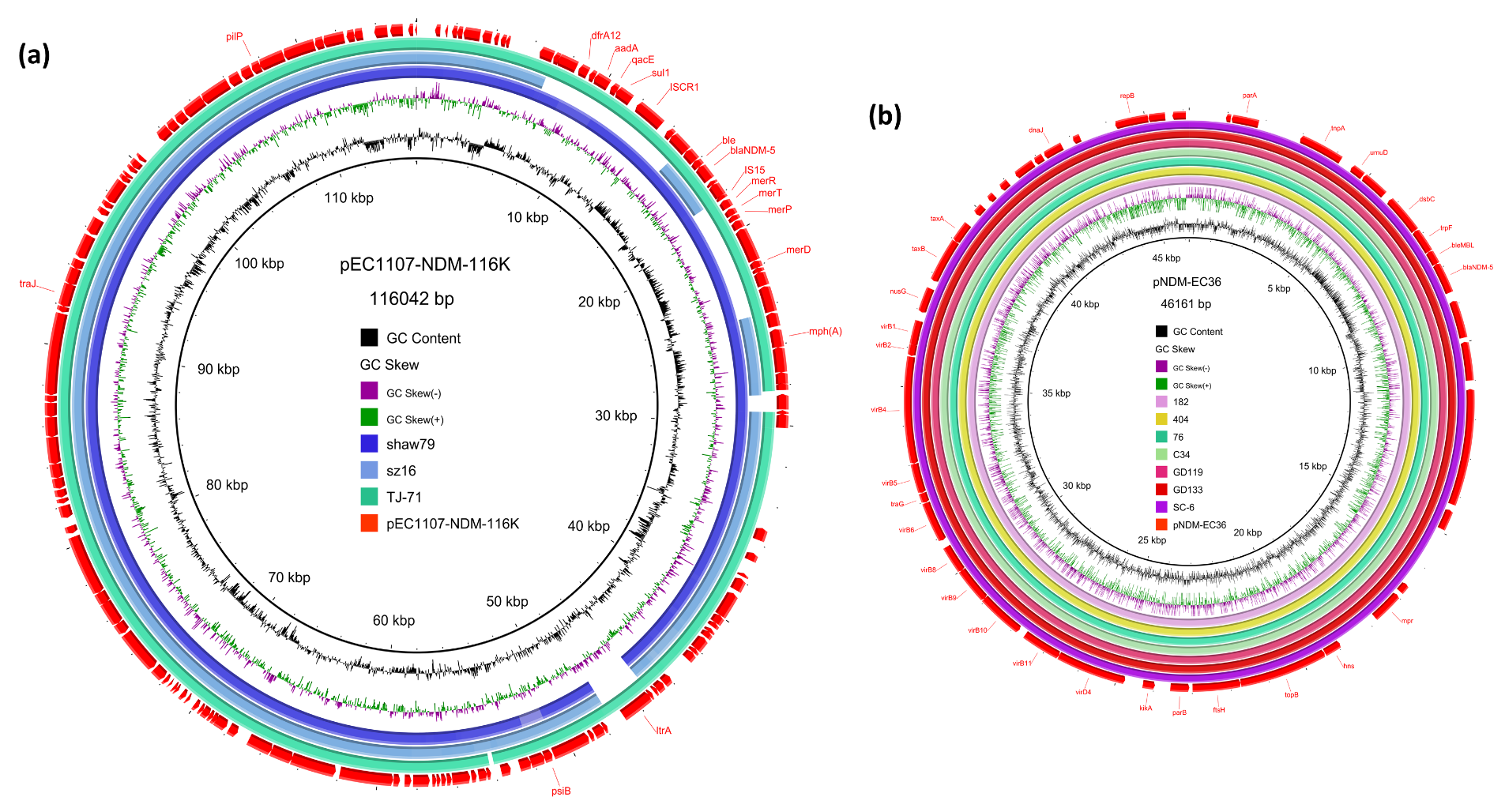


**Supplementary Figure S5. Circular alignment of *bla*_NDM_-bearing plasmids.** (a) pEC1107-NDM-116K-like IncFII plasmid; (b) pNDM-EC36-like IncX3 plasmid.


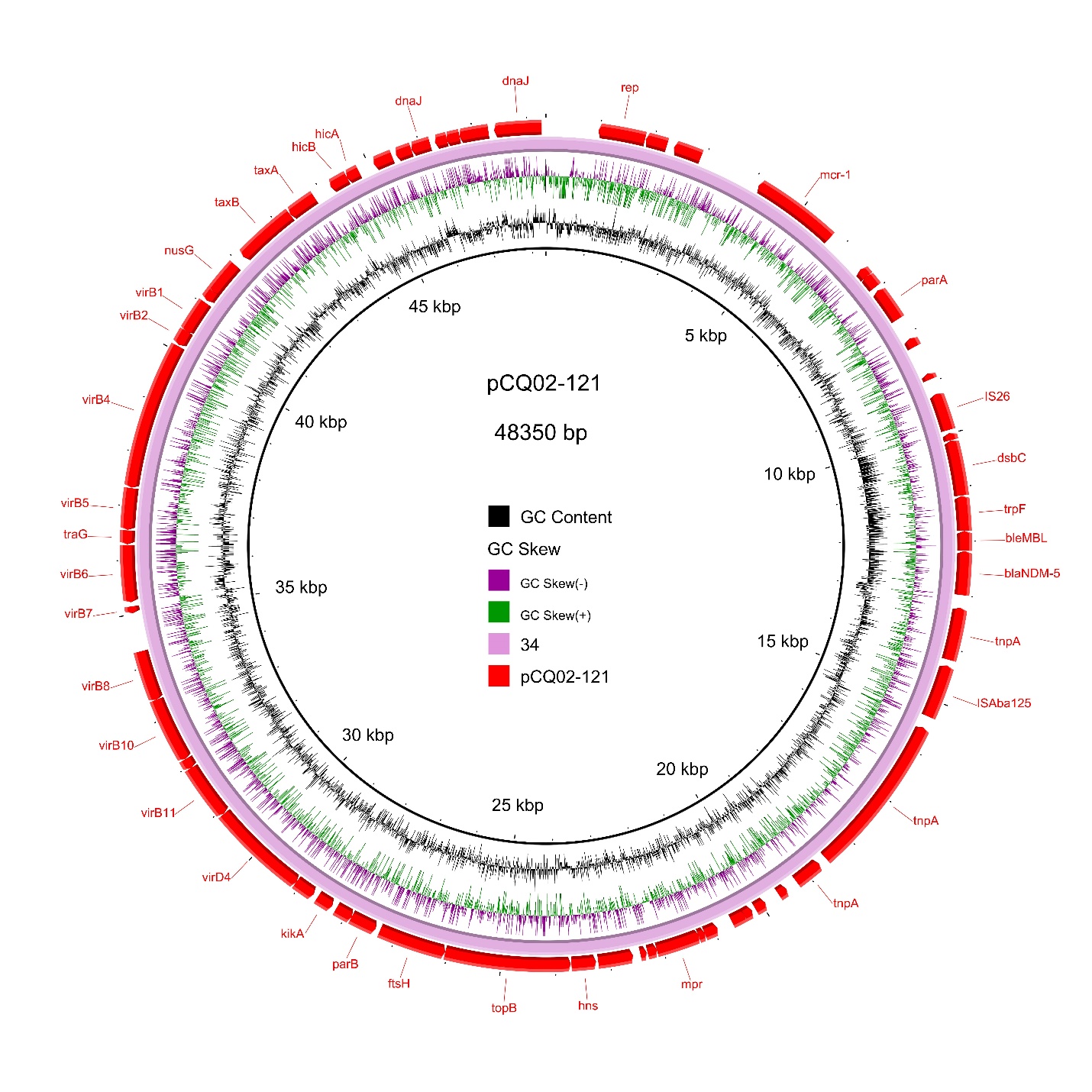


**Supplementary Figure S6. Strain 34 carrying a pCQ02-121-like IncX4 plasmid carrying both the *bla*_NDM_ and *mcr-1* genes.**
